# 121. Evaluation of Multifaceted Antimicrobial Stewardship in Optimizing Antimicrobial Usage in Intraabdominal Infection at a Community Hospital

**DOI:** 10.1093/ofid/ofab466.323

**Published:** 2021-12-04

**Authors:** Tho H Pham, Angela Huang, Scott T Hall, Vanthida Huang

**Affiliations:** 1 Midwestern University College of Pharmacy-Glendale Campus, Glendale, Arizona; 2 HonorHealth John C Lincoln, Phoenix, Arizona; 3 Mayo Clinic Health System-Franciscan Healthcare, La Crosse, Wisconsin; 4 Midwestern University College of Pharmacy - Glendale, Glendale, Arizona

## Abstract

**Background:**

Treatment of intraabdominal infections (IAI) commonly involves broad spectrum antimicrobials based on the severity and etiology of infections as well as the underlying medical conditions. However, the overuse of broad-spectrum agents has driven selection for Gram-negative and -positive resistance, as well as collateral consequences such as *Clostridioides difficile* colitis. We sought to evaluate the utilization of a pharmacy-driven multifaceted antimicrobial stewardship (AMS) intervention to optimize empiric antimicrobial therapy by risk stratification among IAI patients and reduce the number of antibiotic treatment days.

**Methods:**

This is a single-center case observation study in hospitalized adult IAI patients on antimicrobial therapy from Dec 2019-Feb 2020 compared to patients from Dec 2020-Feb 2021 after initiation of AMS with daily prospective audit and feedback. The composite primary outcome is reduction of antibiotic treatment days and de-escalation from broad spectrum antibiotics (fluoroquinolones, piperacillin/tazobactam, and carbapenems) to cephalosporins.

**Results:**

We identified 40 patients each in the baseline (pre-AMS group) and post-AMS group via electronic medical record. Baseline characteristics were well-matched between groups. The majority of patients were diagnosed with community-acquired IAIs such as appendicitis, diverticulitis, and cholecystitis. Fluoroquinolone use as empiric therapy was significantly lower in the post-AMS group vs. pre-AMS group (2.5% vs. 25%, p< 0.001), while non-*Pseudomonas* cephalosporin use was increased (25% post-AMS vs. 0% pre-AMS, p< 0.001). Oral fluoroquinolone use at discharge was significantly decreased in the post-AMS group (p< 0.001). Antibiotic treatment days remained unchanged. There was no statistical difference between the two groups in 30-day mortality, 30-day readmission, relapse, and *C. difficile* colitis.

**Conclusion:**

A multifaceted antimicrobial therapy intervention successfully reduced the use of fluoroquinolones in patients with community-acquired IAI during hospitalization and discharge. No differences in mortality, readmission, or relapse rates were observed.

**Disclosures:**

**All Authors**: No reported disclosures

